# Is Mental Effort Exertion Contagious? A Replication Study

**DOI:** 10.5334/joc.456

**Published:** 2025-07-29

**Authors:** Alessandro Mazza, Ellen Voorrips, Gethin Hughes, Kobe Desender, Eva Van den Bussche, Hans Stuyck

**Affiliations:** 1 KU Leuven, Faculty of Psychology and Educational Sciences, Brain and Cognition, Tiensestraat 102, 3000 Leuven, Belgium; 2 University of Essex, Faculty of Science and Health, Department of Psychology, Wivenhoe Park, CO4 3SQ, Colchester, United Kingdom

**Keywords:** joint Simon task, mental effort contagion, replication, cognitive control

## Abstract

Daily, we perform activities in the presence of others (e.g., office work). While it’s well-established that the mere presence of others can influence our performance, it is less clear whether others’ performance, rather than just their presence, influences us. To address this, we replicated Desender et al.’s (2016) study, *Is mental effort contagious?*, and conducted a second experiment to follow up on our failure to replicate their findings. Desender et al. (2016) used a modified joint Simon task where two participants performed side by side. The manipulated participant completed an easy (mostly congruent trials) and a difficult (mostly incongruent trials) block, while the neutral participant completed two neutral blocks (equal proportion of congruent and incongruent trials). They found that the neutral participant mirrored the manipulated participants’ mental effort, exerting more effort when the latter performed a difficult versus an easy task. In both Experiment 1 (exact replication; *N* = 176) and Experiment 2 (more demanding joint Simon task; *N* = 120), we failed to replicate this result even though the manipulated participants adjusted their mental effort as expected. We identified methodological explanations for this discrepancy in results, such as how conditions were counterbalanced in the original study, which likely produced carry-over effects, and limited visibility of participants’ physiological cues. Moreover, the original study’s effect vanished when re-analyzed with a more robust linear mixed model, suggesting their findings may not have been as reliable as initially thought. Our findings underscore the need for rigorous experimental designs and analyses in psychological research.

## Introduction

In our daily lives, we often find ourselves engaged in activities while being surrounded by others. This can take place in a variety of settings, such as recreational, educational or work-related contexts. This results in sharing one’s space with others while performing a given task. It has been suggested that the mere presence of others can affect our task performance (Guerin, 1986). However, the influence of one’s task performance, rather than the mere presence, on others remains unclear.

The Social Facilitation Theory (Zajonc, 1965) argues that the mere presence of another individual in the vicinity enhances the performance of overlearned or simple tasks (Travis, 1925) but decreases the performance of novel or difficult tasks (Conty et al., 2010; Jun et al., 2017; Miller et al., 2019). One limitation of the Social Facilitation Theory is that it only addresses how the mere presence of other individuals influences task performance (e.g., Bailey et al., 2022; Miller et al., 2019; Rasmussen et al., 2017), not considering the type of actions performed (e.g., Hommel, 2010; Jeannerod, 1999).

Indeed, ideomotor theories suggest that our behaviour is influenced by observing the actions of others. Research has shown that seeing someone else’s behaviour increases the likelihood of the observer carrying out the same behaviour (e.g., Chartrand & Bargh, 1999; McDonough et al., 2022; Schielen et al., 2022; Weingarten et al., 2016). Findings like these suggest that observers actively hold in mind sensorimotor representations of others’ actions that influence the observer’s own behaviour. The Joint Simon task (Sebanz et al., 2003) can be used to understand how individuals mentally represent other individuals’ actions by having them perform a shared version of the traditional Simon task (Simon, 1969). In the traditional Simon task, two types of cues appear individually on either the right or left side of the screen. Participants are instructed to respond to one cue with the left hand and another cue with the right hand. Typically, participants respond faster and more accurately when the spatial location of the cue and the response are congruent (e.g., left-hand cue presented on the left side) compared to incongruent (e.g., left-hand cue presented on the right side). This phenomenon is known as the congruency effect (Leuthold, 2011). In the Joint Simon task, two participants sit next to each other, each assigned a distinct cue color and instructed to respond using only one hand in a go/no-go fashion. Despite performing with a single hand, participants demonstrate a congruency effect: their responses are faster when the spatial location of the cue matches their responding hand (congruent trials) compared to when it does not (incongruent trials). Notably, this congruency effect disappears when the task is performed alone with one hand (Sebanz et al., 2003). The authors argued that when two participants perform the joint Simon task, they create mental representations of their actions and their partner’s actions. This shared representation causes conflict on incongruent trials, like when a single participant performs the traditional Simon task. Therefore, the authors showed that, consistent with ideomotor theories, individuals are influenced also by the actions performed by others. The joint congruency effect has been observed in many other studies (e.g., Kiernan et al., 2012; Tsai et al., 2006; Welsh et al., 2013), although the proposed underlying mechanism has been challenged (e.g., referential coding account; Dolk et al., 2014).

Desender et al. (2016) developed a modified joint Simon task to investigate whether mental effort spills over between participants. One participant (the ‘manipulated participant’) experienced blocks with varying proportions of congruent and incongruent trials: an Easy condition (90% congruent trials) and a Difficult condition (10% congruent trials). In the Easy condition, infrequent incongruent trials increased the congruency effect because participants relied on reactive cognitive control to resolve conflict only when it occurred, leading to poorer performance on these trials (Botvinick et al., 2001). In the Difficult condition, frequent incongruent trials reduced the congruency effect as participants engaged proactive cognitive control, anticipating conflict and improving their performance on these trials. This phenomenon is called the proportion congruency effect (PCE; Bugg & Crump, 2012). Simultaneously, the other ‘neutral participant’ always encountered 50% congruent trials. Both participants fulfilled each role once. A separator screen ensured participants could not observe each other’s trials. To address whether mental effort is contagious, Desender et al. (2016) examined whether the mental effort (i.e., as indexed by the size of the congruency effect) exerted by the neutral participant was influenced by the mental effort exerted by the manipulated participant.

They found that the congruency effect displayed by the neutral participant mirrored the congruency effect displayed by the manipulated participant for both the Easy and Difficult condition and concluded that mental effort exertion is contagious. This means that the neutral participant adapted their effort, akin to the mental effort invested by the manipulated participant. Desender et al. (2016) argued that this effect might be due to the neutral participant perceiving subtle cues in the manipulated participant that convey their degree of exerted mental effort, such as subtle changes in posture (see Friedman & Elliot, 2008). Considering that neutral participants had an equal proportion of congruent and incongruent trials and could not see the trials of the manipulated participant, they had no explicit reason to adapt their mental effort exertion across blocks of trials.

Compared to similar prior studies (e.g., Sebanz et al., 2003; Tsai et al., 2006), participants had little information on their adjacent participant’s activity, as they couldn’t see each other’s trials. Moreover, participants only responded with one hand and only saw the stimuli they had to respond to. Consequently, the task likely required minimal mental effort adaptation. The fact that the mental effort exerted by the manipulated participant was likely subtle, combined with the limited information available for the neutral participant to infer this effort, renders these results particularly impressive. However, the observed effects were large ( \[
\eta_{p}^{2}
\] = 0.15) with a relatively small sample size (*N* = 38), which can inflate the risk of Type I errors (i.e., a false positive) and overestimate effect sizes (Lakens, 2022). This raises whether the observed effect is perhaps overestimated or coincidental (Lakens, 2022).

**Figure 1:**
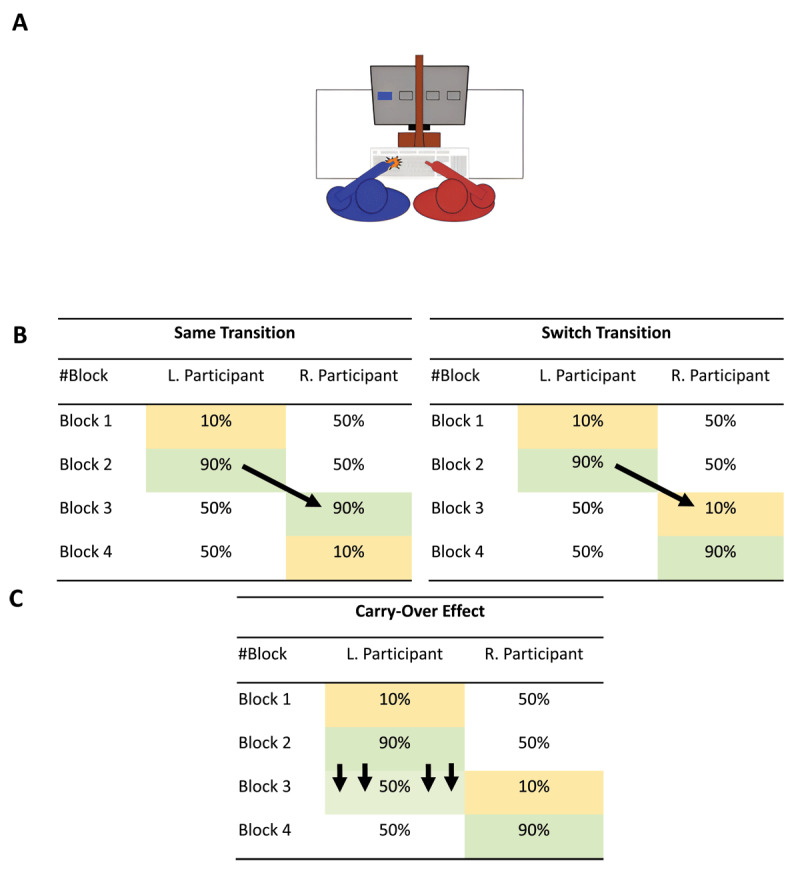
The Experimental Setup and Design. *Note*. Panel A provides an example of the setup for a dyad of two participants. The participant on the right responds with their right hand, and the participant on the left with their left hand. Panel B represents an example of the same and switch transition types between blocks 2 and 3, which were counterbalanced across participants. Same Transition, the transition between blocks 2 and 3 entails the same manipulated proportion congruency for the manipulated participant in block 2 (90%) and the neutral participant becoming the manipulated one in block 3 (i.e., 90%). Switch Transition, the transition between blocks 2 and 3 entails a different manipulated proportion congruency for the manipulated participant in block 2 (90%) and the neutral participant becoming the manipulated one in block 3 (10%). L. Participant, is the left participant who is initially manipulated and transitions to becoming the neutral one in blocks 3 and 4. R. Participant, is the right participant who is initially the neutral one that transitions to becoming the manipulated one in blocks 3 and 4. The order of either the left or right participants starting as the neutral or manipulated one was also counterbalanced across participants. Panel C marks the point in the sequence of blocks of trials where a carry-over effect could occur (i.e., between blocks 2 and 3), where the mental effort exerted by the manipulated participant in block 2 might carry over to block 3 when that participant becomes the neutral one.

One drawback in the study of Desender et al. (2016) is that they only used one transition type between blocks 2 and 3 instead of counterbalancing the different transition orders (see Figure 1B for the different types of transition). Each participant completed four blocks: two manipulated and two neutral. Between the second and third blocks, participants transitioned unknowingly from being the manipulated to being the neutral participant or vice versa. In the article, the authors state that this transition was always switched. That is, from an Easy condition (90% congruent trials) in block 2 to a Difficult condition for the previously neutral participant (10% congruent trials) in block 3 or vice versa. However, the transition from block 2 to block 3, in fact, was always the same (i.e., Easy to Easy or Difficult to Difficult). Note that the paper incorrectly reported that there was always a switch transition between blocks 2 and 3 (discovered after a discussion with the lead author). Thus, potential carry-over effects were not accurately controlled for as reported. Carry-over effects might represent an issue in repeated measures within-subject designs as the response performance by participants on later blocks might depend on their performance in previous blocks (see Figure 1C; Brooks, 2012). As such, a more optimal design should counterbalance the transition types (i.e., switch transition versus same transition between blocks 2 and 3).

Considering the concerns addressed above and the current replication crisis in psychology (Wiggins & Christopherson, 2019), a replication of Desender et al. (2016) is essential. To address the potential lack of power in the original study, we aimed for a larger sample size (see Participants section), which minimizes the risk of a Type II error and more robustly determines whether the exertion of mental effort is, indeed, contagious from one individual to another. Additionally, we used linear mixed models (i.e., LMM) alongside the original ANOVA analysis to reduce the risk of a Type I error. LMMs achieve this by taking random variability due to participants, block, and/or trial types into account (Barr et al., 2013). Our replication effort involved two experiments. Experiment 1 entails an exact replication of study 2 of Desender et al. (2016) described above.^1^

Crucially, we included the two possible types of transitions between blocks 2 and 3 (switch or same) and counterbalanced the type of transitions across participants. Given that we could not replicate the original findings, we next performed Experiment 2, in which we increased the task difficulty by having each participant conduct a full Simon task. This modification aimed at increasing the mental effort required, as the spatial location of the cues and responses can now vary for both hands. If the effect originally observed truly reflects the contagion of mental effort, it should be more pronounced under more demanding conditions. Additionally, to address potential carry-over effects, Experiment 2 included a wash-out block between blocks 2 and 3 where both participants performed a neutral condition.

For both Experiments, we hypothesized that the congruency effect of the neutral participant in terms of reaction times (i.e., RTs) and error rates will be reduced when the manipulated participant is performing a Difficult condition, compared to an Easy condition. This would prove that mental effort is contagious (cf. Desender et al., 2016). Before being able to demonstrate that mental effort is contagious, it is crucial that the manipulated participant adjusts their level of mental effort based on whether they are in an Easy or Difficult condition. Therefore, we expect the manipulated participant to show a reduced congruency effect in the Difficult compared to the Easy condition. We preregistered Experiment 1 (see https://osf.io/6n4yf) and Experiment 2 (see https://osf.io/68uj3), outlining the hypotheses, sample size, and statistical analysis. Any deviations from the preregistration can be found in Supplementary file 1: Appendix A. All data, code, and Supplementary file 1 appendices can be accessed on the Open Science Framework (i.e., https://osf.io/486vn/).

## Experiment 1

### Method

#### Participants

A convenience sample of 176 undergraduates was recruited, of which 148 were female. For their participation, they received course credit. All participants reported having normal or corrected-to-normal vision. The mean age was 18.5 years (range 17–35, *SD* = 2.1).^2^ Participants were divided into dyads of two participants, resulting in 88 dyads. A minimum a priori sample size of 128 participants was determined to detect a medium effect size (i.e., \[
\eta_{p}^{2}
\] = 0.06) with 80% power and an alpha level of .05 (Campbell & Thompson, 2012). We chose not to rely on the large effect size (i.e., \[
\eta_{p}^{2}
\] = 0.15) reported by Desender et al. (2016), as it was based on a relatively small sample size (*N* = 38), likely leading to an overestimation. Instead, we aimed for a more conservative medium effect size, which is generally considered valid in the field of cognitive psychology (Brysbaert & Stevens, 2018). The current sample (i.e., *N* = 176) allowed us to detect a small-to-medium effect size (i.e., \[
\eta_{p}^{2}
\] = 0.04) with a statistical power of 80% and an alpha level of .05. Before the onset of the experiment, all participants provided written informed consent. At the end of the experiment, each participant was asked to score their level of acquaintance with the other participant of the dyad on a scale from 0 (not at all) to 5 (very well). The mean score of acquaintance was *M* = 1.61 (range = 0–5, *SD* = 1.73).

#### Material

The experiment was developed in E-prime (Schneider et al., 2002) and presented on a 20-inch Dell LCD Monitor.

#### Experimental Procedure

Participants were matched in dyads via the online platform through which they enrolled for the experiment. A dyad was formed if two participants enrolled in the same time slot. In the lab, the two participants forming a dyad were seated in front of one computer screen. A separator screen split the computer screen in half (see Figure 1A), such that each participant in a dyad could only see the stimuli that they themselves had to respond to. Participants used the same keyboard to record their response. The stimuli presented in the task were of four different colours: blue, yellow, green, and orange. Each participant from the dyad was assigned two cue colours to which they had to respond. The colours were presented as square shapes (i.e., 3.5° wide and 3.5° high) on a black background and only appeared on the side of the screen of the participant assigned to those colours. All colour combinations were counterbalanced across participants. The participant on the left was instructed to respond to the target colours with their left index finger by pressing the letter ‘D’. The participant on the right was instructed to respond to the target colours with their right index finger by pressing the letter ‘K’. First, a fixation cross was presented for 800 ms at the centre of the screen’s Y-axis but at 25% of the screen’s X-axis for the left participant and at 75% of the screen’s X-axis for the right participant. This was followed by a single square-shaped cue colour per trial, presented at the centre of the screen’s Y-axis, but at either 10% (i.e., the left side) or 40% (i.e., the right side) of the screen’s X-axis for the participant on the left or at either 60% (i.e., the left side) or 90% (i.e., the right side) of the screen’s X-axis for the participant on the right. The cue colour disappeared after a response was made or after maximally 3000 ms. There was an inter-trial interval of 1000 ms. The dyad was first presented with 16 practice trials during which the separator screen was removed to ensure participants were aware that they were both performing the same task alongside each other. Feedback on accuracy was provided during these practice trials. After the practice trials, the experiment leader checked if the participants understood the task and stressed that they could not communicate with each other for the remainder of the experiment. For the experimental trials, the separator screen was placed between the participants. The experiment progressed through four blocks of 160 trials which each existed of 80 trials presented to each participant. Therefore, the total amount of experimental trials was 640 trials per dyad (i.e., 2 participants _×_ 80 trials _×_ 4 blocks).

#### Experimental Design

In Figure 1B, the experimental design is depicted. The experimental design of Desender et al. (2016) consisted of two manipulated conditions and one neutral condition. The two manipulated conditions consisted of an Easy and a Difficult condition. In the Easy condition, 90% of the trials were congruent—that is, the target cue’s position on the screen (e.g., left side) aligns with the participants’ response hand (e.g., left hand). In the Difficult condition, only 10% of the trials were congruent. The Neutral condition always consisted of 50% congruent trials. There were four blocks of trials. In the first two blocks of trials (i.e., blocks 1 and 2), one participant in the dyad was the manipulated participant, and the other was the neutral participant. The manipulated participant could receive an Easy condition in block 1 and a Difficult condition in block 2 or vice versa, and the neutral participant would receive the Neutral condition in the first two blocks. In blocks 3 and 4, the participants in the dyad switched conditions. Namely, the neutral participant in blocks 1 and 2 became the manipulated participant in blocks 3 and 4, and vice versa for the manipulated participant in blocks 1 and 2. The order of the manipulated conditions in blocks 1 and 2 and blocks 3 and 4 was counterbalanced across participants (e.g., block 1 = Difficult condition and block 2 = Easy condition or block 1 = Easy condition and block 2 = Difficult condition). Finally, a critical design improvement compared to Desender et al. (2016) is that we included both possible transition types when transitioning from block 2 to block 3. Specifically, we fully counterbalanced the block2-to-block3 transitions. When the neutral and the manipulated participants swapped roles between blocks 2 and 3, half of these pairs experienced the same transitions between these blocks (Easy→Easy or Difficult→Difficult), and the other half experienced switch transitions (Easy→Difficult or Difficult→Easy). See Figure 1B for an example of both conditions.

#### Statistical Analysis

Participants exceeding an error rate of 20% were excluded from the analysis. However, on average, only 0.18% errors were made per participant. Thus, no participant was excluded based on the error rates. Moreover, the number of errors was so low that there was not enough data to perform a proper statistical analysis. Next, trials were excluded from the analysis if no response was provided or if their RT was faster than 100 ms, which is often considered a very fast response due to the participant’s anticipation of seeing a target stimulus (Rubichi et al., 2000; Stürmer et al., 2002). After this, the remaining trials were excluded if they exceeded ±2.5*SD* of the mean calculated per participant. These exclusion criteria led to the exclusion of 1419 trials (2.52% of all trials), leaving 55001 correctly solved trials for the statistical analysis.

To test our main hypothesis, we first conducted the same analysis as Desender et al. (2016). Specifically, a 2 × 2 repeated measures ANOVA was conducted on the median RTs of the participant performing the Neutral condition (i.e., the neutral participant), with two within-subject factors: congruency with two levels (congruent and incongruent) and block type of the manipulated participant with two levels (Easy condition and Difficult condition). Posthoc tests were only conducted for significant interaction effects, as the main effects involved only two levels, making further comparisons unnecessary. For significant interaction effects, congruency was pairwise contrasted, conditioned on the block type of the manipulated participant (i.e., congruent vs. incongruent within the Easy condition and Difficult condition). Cohen’s *d* effect sizes accompanied all pairwise contrasts for both significant main and interaction effects. We interpreted Cohen’s *d* as 0.20 = small, 0.50 = medium, and 0.80 = large (Cohen, 1988).

To further substantiate the results of the repeated measures ANOVA specified above, a linear mixed model (i.e., LMM) was built with the single trial RTs of the participant performing the Neutral condition (i.e., neutral participant) as the outcome variable. LMM is a more powerful type of statistical analysis than ANOVA (Barr et al., 2013). It can treat participants, the type of block of trials (i.e., Easy and Difficult), and the type of trial (i.e., congruent and incongruent) as crossed random effects, taking between-participant, between-block-type and between-trial-type variability into account (Baayen et al., 2008). This reduces the likelihood of a Type I and II error (de Melo et al., 2022; Matuschek et al., 2017). The fixed effects predicting the RTs in the LMM were congruency (i.e., congruent and incongruent) and block type of the manipulated participant (i.e., Easy condition and Difficult condition). Satterthwaite approximation method was used to determine the significance of the fixed effects. Each LMM included a random intercept for the participants. Next, in a forward fashion, we stepwise added to the random structure random slopes for (1) congruency, (2) the block type of the manipulated participant, (3) their combination, and (4) their interaction (Barr et al., 2013; Bates et al., 2015). The LMMs that converged were subsequently compared using likelihood ratio tests to determine the LMM with the best fit (Brown, 2021). To assess the significance of the main and interaction effects, we compared the full LMM, with all fixed effects included, to three reduced models that either excluded one of the main effects or the interaction effect. Subsequently, a likelihood ratio test (χ^2^) was used to measure if the full model explained more residual variance than the reduced models. Hence, finding a statistically significant effect implied a main or interaction effect (Zeileis & Hothorn, 2002). Posthoc tests were only conducted for significant interaction effects like what is described for the ANOVA analysis.

We also analysed the manipulated participants to examine whether manipulating the proportion of congruent and incongruent trials in the Easy and Difficult conditions had the aimed-for effect on the mental effort exertion of the manipulated participants. For this analysis, we conducted the same procedure specified above with the median RTs (i.e., for the ANOVA procedure) and single trial RTs (i.e., for the LMM procedure) of the correctly solved trials of the manipulated participants as the outcome variable.

The statistical analysis was conducted using R software (R Core Team, 2022). For a list of the packages used, see Supplementary file 1: Appendix B. The full models for both ANOVA and LMM analyses can be found in Supplementary file 1: Appendix C. The LMM analysis will only be reported if it deviates from the ANOVA analysis, else it will be reported in Supplementary file 1: Appendix D.

### Results

#### ANOVA with RT as the Outcome

**Neutral Participants.** A 2 × 2 repeated measures ANOVA was used to analyse median RTs of the correctly solved trials of the neutral participant with congruency (i.e., congruent and incongruent), and block type of the manipulated participant (i.e., Easy and Difficult condition) as independent variables. This analysis only revealed a significant main effect of congruency, *F*(1, 175) = 173.68, *p* < .001, with faster RTs on congruent trials (*M* = 323 ms) than incongruent trials (*M* = 340 ms), Cohen’s *d* = 0.28 (95% CI [0.24, 0.31]). The main effect of block type of the manipulated participant and the interaction effect were not significant, *p* = .986 and *p* = .230, respectively.

**Manipulated Participants.** The same ANOVA analysis was conducted on median RTs of correct trials of the manipulated participants. This analysis revealed a main effect of congruency, *F*(1, 175) = 64.58, *p* < .001, with faster RTs on congruent trials (*M* = 329 ms) compared to incongruent ones (*M* = 344 ms), Cohen’s *d* = 0.26 (95% CI [0.19, 0.33]). Moreover, the analysis revealed a significant main effect of block type of the manipulated participant, *F*(1, 175) = 6.30, *p* = .013, with slower RTs during the Easy condition (339 ms) compared to the Difficult condition (334 ms), Cohen’s *d* = 0.09 (95% CI [0.02, 0.16]). Crucially, a significant interaction effect was found, *F*(1, 175) = 23.02, *p* < .001. Posthoc tests revealed a congruency effect when the manipulated participants performed an Easy condition, with faster RTs for congruent trials (*M* = 326 ms) than incongruent trials (*M* = 351 ms), *t*(175) = 8.62, *p* < .001, Cohen’s *d* = 0.56 (95% CI [0.46, 0.67]). In contrast, no significant congruency effect was observed when the manipulated participants performed the Difficult condition, *p* = .077. This result demonstrates the expected PCE with a larger congruency effect in the Easy versus Difficult condition.

**Figure 2:**
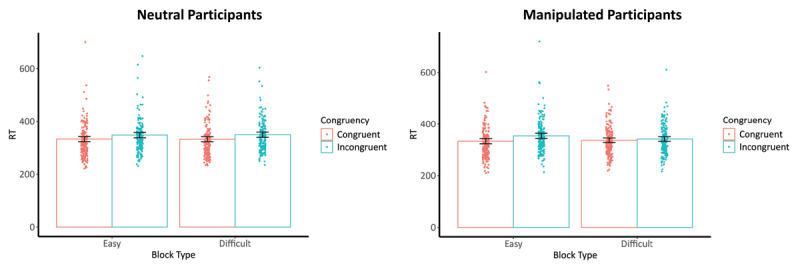
RTs on (In)Congruent Trials of the Neutral and Manipulated Participants Depending on Difficulty of the Condition of the manipulated participant. *Note*. RT, reaction time; Easy block, block where the manipulated participant received 90% congruent trials; Difficult block, block where the manipulated participant received 10% congruent trials; left graph are the RTs on the (in)congruent trials of the neutral participants depending on the difficulty of the condition of the manipulated participants; right graph are the RTs on the (in)congruent trials of the manipulated participants depending on the difficulty of the condition they performed; Dots are the participants’ median RTs depending on the levels of congruency within each block type; lines on top of the bars are the 95% confidence intervals.

The LMM analyses demonstrated results similar to those of the abovementioned ANOVA analyses (see Supplementary file 1: Appendix D). Figure 2 illustrates the results of these analyses.

#### Exploratory Analyses

The main analyses described above could not replicate the results of Desender et al. (2016). To address the possibility that the null result was due to carry-over effects resulting from participants switching roles between the first two and the last two blocks, we conducted an exploratory analysis restricted to the first two blocks—where no prior trials could have induced such an effect. Moreover, as Desender et al. (2016) only used the same transition type for the transition from blocks 2 to 3, we decided to conduct an additional exploratory analysis where we ran the same analysis as outlined above but separately for the same transition type (i.e., *N* = 88) and the switch transition type (i.e., *N* = 88). Lastly, given our failure to replicate and the large effect size Desender et al. (2016) reported (i.e., \[
\eta_{p}^{2}
\] = 0.15) with a relatively small sample (i.e., *N* = 38), which increases the risk of Type I error (Lakens, 2022), we also re-analyzed their original data using the more robust LMM approach. The full models of each analysis can be found in Supplementary file 1: Appendix E.^3^

**Blocks 1 and 2**. For this analysis, we conducted an LMM on the RTs as the outcome variable, as this is a more robust analysis than the ANOVA. For the neutral participants, only a main effect of congruency was observed, χ²(1) = 92.87, *p* < .001, with faster congruent (*M* = 322 ms) than incongruent trials (*M* = 338 ms), Cohen’s *d* = 0.25 (95% CI [0.20, 0.30]). The main effect of block type and the interaction effect were not significant, *p* = .693 and *p* = .754, respectively. For the manipulated participants, the analysis revealed a main effect of congruency (i.e., *p < .001*, see full model), and, more crucially, a significant interaction effect between congruency and block type, χ²(1) = 13.28, *p* < .001. While in the Easy condition there was a congruency effect with faster congruent (*M* = 333 ms) than incongruent trials (*M* = 359 ms), *t*(89) = 6.33, *p* < .001, Cohen’s *d* = 0.40 (95% CI [0.27, 0.53]), this congruency effect was only marginally significant in the Difficult condition, *M* = 338 ms vs. *M* = 344 ms, *t*(89) = 1.98, *p* = .051, Cohen’s *d* = 0.09 (95% CI [0.00, 0.18]). This result shows the expected PCE for the manipulated participant. The main effect of block type was not significant, *p* = .610. This analysis confirms that, even when limiting our data to the first two blocks (before any possible carry-over effects can occur), we still find no evidence of effort “contagion” from manipulated to neutral participants—despite the manipulated participants showing the expected PCE.

**Same Transition type**. In the repeated measures ANOVA analysis with the RT of the neutral participant as the outcome variable, only a main effect of congruency was observed, *F*(1, 87) = 91.84, *p* < .001, with faster RTs on congruent trials (*M* = 319 ms) than incongruent ones (*M* = 334 ms), Cohen’s *d* = 0.31 (95% CI [0.25, 0.37]). The main effect of block type and the interaction effect were not significant, *p* = .217 and *p* = .659. The same ANOVA but with the RT of the manipulated participants as the outcome variable similarly only showed a main effect of congruency, *F*(1, 87) = 26.83, *p* < .001, with faster RTs on congruent trials (*M* = 328 ms) than incongruent ones (*M* = 340 ms), Cohen’s *d* = 0.25 (95% CI [0.14, 0.37]). The main effect of block type and the interaction effect were not significant, *p* = .711 and *p* = .584. The same set of analyses with LMMs were in line with these findings. Again, we could not observe a contagious mental effort like what was found in the main analysis. However, we could not observe an adaptation of mental effort exertion by the manipulated participant depending on the block type they received. This limits the conclusions that can be drawn from this analysis as mental effort adaption by the manipulated participants is necessary for it to spill over from the manipulated to the neutral participant.

**Figure 3:**
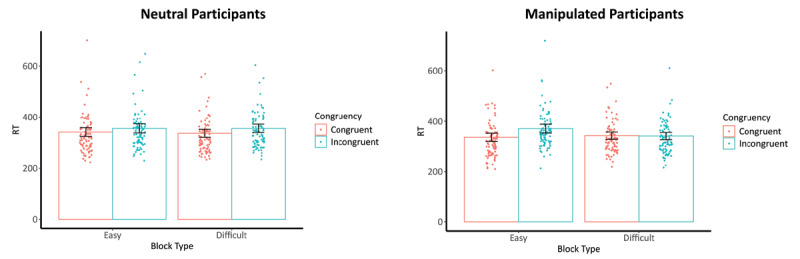
RTs on (In)Congruent Trials of the Neutral and Manipulated Participants for the Switch Transition Type. *Note*. RT, reaction time; Easy block, block where the manipulated participant has 90% congruent trials; Difficult block, block where the manipulated participant has 10% congruent trials; left graph are the reaction times on the (in)congruent trials of the neutral participants depending on the difficulty of the condition of the manipulated participants; right graph are the reaction times on the (in)congruent trials of the manipulated participants depending on the difficulty of the condition they performed; Dots are the participants’ median RTs depending on the levels of congruency within each block type; lines on top of the bars are the 95% confidence intervals.

**Switch Transition Type**. The repeated measures ANOVA analysis with RT of the neutral participant as the outcome variable revealed a main effect of congruency (i.e., *p* < .001; see full model) and, more crucially, an interaction effect was observed, *F*(1, 87) = 4.30, *p* = .041. Critically, post hoc tests revealed a congruency effect with faster RTs on congruent (*M* = 330 ms) than incongruent trials (*M* = 345 ms) when the manipulated participant performed an Easy condition, *t*(87) = 5.76, *p* < .001, Cohen’s *d* = 0.21 (95% CI [0.14, 0.28]). Although a similar congruency effect was observed with faster RTs on congruent trials (*M* = 325 ms) than incongruent ones (*M* = 345 ms) when the manipulated participant performed the Difficult condition, *t*(87) = 10.60, p < .001, Cohen’s *d* = 0.31 (95% CI [0.25, 0.37]), the congruency effect was less pronounced in the Easy than Difficult condition, 5 ms versus 10 ms, respectively. Thus, we unexpectedly observed a slightly larger congruency effect in the Difficult versus Easy condition. This result is illustrated in Figure 3. The main effect of block type was not significant, *p* = .386. The same ANOVA analysis but with the RT of the manipulated participants as the outcome variable revealed a main effect of congruency (i.e., *p* < .001; see full model), a main effect of block type (i.e., *p* = .001; see full model) and, more crucially, a significant interaction effect, *F*(1, 87) = 109.76, *p* < .001. Posthoc tests revealed a congruency effect with faster RTs for congruent trials (*M* = 325 ms) than incongruent ones (*M* = 364 ms) when the manipulated participants performed an Easy condition, *t*(348) = 3.99, *p* < .001, Cohen’s *d* = 0.57 (95% CI [0.28, 0.89]). However, no significant congruency effect was found when the manipulated participants performed the Difficult condition, *p* = .659. Unlike the opposite PCE observed for the neutral participants, the manipulated participants demonstrated the expected PCE with a larger congruency effect in the Easy versus Difficult condition. The same set of analyses with the LMMs provided similar results.

To follow up on this unexpected result, we conducted the same LMM analysis on the neutral and the manipulated participants’ RTs but now separately for blocks 1 and 2 on the one hand and blocks 3 and 4 on the other hand. We only reran the LMM as this is a more robust analysis than the ANOVA. This analysis allowed us to determine if the observed unexpected effect resulted from a carry-over effect. If the effect was present in blocks 3 and 4 and not blocks 1 and 2—where a carry-over effect could not occur as there are no preceding blocks—this would provide evidence that the observed patterns for the neutral participants are likely due to carrying-over the mental effort they exerted in block 2 to block 3 (i.e., the neutral participant who was previously the manipulated one in block 2), irrespective of what the manipulated participant performed in block 3.

Regarding the analysis of blocks 1 and 2, for the neutral participants, there was only a main effect of congruency, χ^2^(1) = 28.86, *p* < .001, displaying faster RTs on congruent (*M* = 323 ms) than incongruent trials (*M* = 341 ms), Cohen’s *d* = 0.24, (95% CI [0.16, 0.31]). The main effect of block type and the interaction effect were not significant, *p* = .527 and *p* = .417, respectively. For the manipulated participants, there was a main effect of congruency (i.e., *p* < .001; see full model) and block type (i.e., *p* < .001; see full model). More crucially, there was a significant interaction effect, χ^2^(1) = 45.35, *p* < .001. While in the Easy condition there was a congruency effect with faster congruent (*M* = 339 ms) than incongruent trials (*M* = 377 ms), *t*(130) = 6.68*, p* < .001, Cohen’s *d* = 0.55 (95% CI [0.38, 0.71]), this congruency effect was not significant in the Difficult condition, p = .965. Regarding the analysis of blocks 3 and 4, for the neutral participants, the same LMM analysis revealed a main effect of congruency (i.e., *p* < .001; see full model) and, importantly, a marginally significant interaction effect, χ^2^(1) = 3.67, *p* = .055. Post hoc tests revealed a congruency effect with faster RTs on congruent trials (*M* = 358 ms) than incongruent ones (*M* = 370 ms) when the manipulated participant performed an Easy condition, *t*(149) = 3.84, *p* < .001, Cohen’s *d* = 0.14, (95% CI [0.07, 0.21]). Although a similar congruency effect was observed with faster RTs on congruent trials (*M* = 352 ms) than incongruent ones (*M* = 371 ms) when the manipulated participant performed the Difficult condition, *t*(149) = 6.40, *p* < .001, Cohen’s *d* = 0.24 (95% CI [0.16, 0.31]), the difference in RTs between congruent and incongruent trials was less pronounced in the Easy than Difficult condition, 11.7 ms versus 19.6 ms, respectively. The main effect of block type was not significant, *p* = .174. For the manipulated participants, there was a main effect of congruency (i.e., *p* < .001; see full model) and block type (i.e., *p* < .001; see full model). More crucially, there was a significant interaction effect, χ^2^(1) = 26.64, *p* < .001. While in the Easy condition there was a congruency effect with faster congruent (*M* = 333 ms) than incongruent trials (*M* = 364 ms), *t*(388) = 5.87, *p* < .001, Cohen’s *d* = 0.38 (95% CI [0.25, 0.51]), this congruency effect was not significant in the Difficult condition, *p* = .546. This finding demonstrates that, although the manipulated participants showed the expected PCE in the first two and last two blocks, the opposite of the expected PCE was observed for the neutral participants, but only in blocks 3 and 4. Therefore, this is more likely to reflect a simple carry-over effect, as this finding only occurred in these two last blocks, where carry-over was possible.

**Linear Mixed Model on the Original Data of Desender et al**. (2016). An LMM was constructed with the RT of correctly solved trials of the neutral participant as the outcome variable and congruency (i.e., congruent and incongruent), block type of the manipulated participant (i.e., Easy and Difficult condition), and their interaction as the fixed effects. There was only a main effect of congruency, χ^2^(1) = 9.58, *p* = .002, displaying faster RTs on congruent trials (*M* = 383 ms) than incongruent ones (*M* = 402 ms), Cohen’s *d* = 0.10 (95% CI [0.04, 0.16]). The main effect of block type and the interaction effect were not significant, *p* = .660 and *p* = .450, respectively. The same LMM analysis was performed but now with the RT of the manipulated participants as the outcome variable. This revealed a significant interaction effect, χ^2^(1) = 12.20, *p* < .001. Posthoc tests revealed a congruency effect with faster RTs on congruent trials (*M* = 391 ms) than incongruent ones (*M* = 427 ms) when the manipulated participant was performing an Easy condition*, t*(176) = 3.19, *p* = .002, Cohen’s *d* = 0.34 (95% CI [0.26, 0.42]). Contrarily, no significant congruency effect was observed when the manipulated participant was performing a Difficult condition, *p* = .137. Congruency and block type main effects were not significant, *p* = .252 and *p* = .114, respectively. This result shows that even though the manipulated participant adjusted their mental effort exertion based on the difficulty of the conditions, there was no contagion on the mental effort exerted by the neutral participant. This contradicts the ANOVA results reported by Desender et al. (2016). As LMM takes between-participant, between-block-type and between-trial-type variability into account (Baayen et al., 2008), this likely explained away some variability that drove the effect observed in the original study. This reveals that the result observed in their study was likely due to a Type I error.

### Interim Discussion

In Experiment 1, we aimed to replicate Desender et al.’s (2016) study on mental effort contagion using their modified joint Simon task. Manipulated participants performed Easy or Difficult blocks (low vs. high effort), while neutral participants performed neutral blocks to assess whether their mental effort adjusted to the manipulated participants’. Contrary to Desender et al. (2016), we found no evidence that neutral participants adjusted their mental effort to match that of the manipulated participants, although the manipulated participants adapted their effort based on block difficulty. Exploratory analyses of same and switch transitions between blocks 2 and 3 revealed intriguing results. While the same transition yielded no clear effects, the switch transition showed opposing PCEs: neutral participants displayed a reversed PCE based on manipulated participants’ block difficulty, whereas manipulated participants showed the expected PCE. Follow-up analyses suggest these patterns arose from carry-over effects, where effort exerted by the manipulated participants in block 2 carried over into their performance as neutral participants in block 3.

We also re-analyzed Desender et al.’s (2016) original data using a more robust LMM analysis than their ANOVA analysis. Again, we failed to replicate their results. Our findings suggest that the initial results of Desender et al. (2016) might be due to either the inflating influence of a carry-over effect or a Type I error.

An alternative explanation for the frailty of the effort contagion effect is that participants may not have experienced the task as sufficiently effortful. With near-ceiling accuracy in the current and original study (0.4% errors), changes in effort exertion were likely subtle and hard for neutral participants to detect, limiting the conclusions that can be drawn from Experiment 1. To address this, Experiment 2 increased task demands by having each participant perform a full traditional Simon Task, known to elicit strong (proportion) congruency effects.

## Experiment 2

### Method

#### Participants

A convenience sample of 120 undergraduates was recruited, of which 105 were female. For their participation, they received course credit. All participants reported having normal or corrected-to-normal vision. The mean age was 18.6 years (range 18–27, *SD* = 1.4). Participants were randomly divided into dyads of two participants, resulting in 60 dyads. A minimum a priori sample size was determined following the same rationale as in Experiment 1, entailing 120 participants. Such a sample size would allow us to detect a medium effect size (i.e., \[
\eta_{p}^{2}
\] = 0.06) with a power of 80% and an alpha level of .05 (Campbell & Thompson, 2012). Before the onset of the experiment, all participants provided written informed consent. At the end of the experiment, each participant was asked to score their level of acquaintance with the other participant of the dyad on a scale from 0 (not at all) to 5 (very well). The mean acquaintance score was *M* = 2.34 (range = 0–5, *SD* = 1.99).

#### Material

The experiment was developed in PsychoPy for Windows (Peirce et al., 2019) and run on a Dell with a 20-in LCD Monitor.

#### Experimental Procedure

The experimental setup is similar to Experiment 1 (see Figure 1A), except the setup allows each participant to perform a full traditional Simon task. Below, we only list the deviations from the experimental setup of Experiment 1. Each participant was assigned two colours in the form of a circle. Each of the two circle-shaped colours (i.e., 2° diameter) would only appear on each participant’s side of the screen. The colours were: blue, purple, green, and orange. The circle-shaped colours were presented on a grey background. The colour combinations were counterbalanced across participants. The participant on the left was instructed to use the left index finger to press the letter ‘A’ and the right index finger to press the letter ‘S’. The participant on the right was instructed to press the letter ‘J’ with their left index finger and the letter ‘K’ with their right index finger. Throughout the experiment, they were instructed to keep their finger on their respective keys. Subsequently, each colour was then assigned to one of the two keys attributed to the participant (e.g., the left participant is assigned blue and purple, and they must press the ‘A’ key when they see a blue circle and ‘S’ when they see a purple circle). Before the experiment, 24 practice trials were presented with feedback on the correctness of participants’ responses.

#### Experimental Design

All was identical to Experiment 1, except for a wash-out block of 72 trials (i.e., 36 trials/participant) inserted between blocks 2 and 3, consisting of 50% congruent and 50% incongruent trials. This was implemented to counteract carry-over effects that might occur when participants switch from being the manipulated participant in block 2 to becoming the neutral participant in block 3 and vice versa. This amounts to 5 blocks comprising 712 trials (i.e., block 1, block 2, wash-out block, block 3 and block 4). Furthermore, only the same switch type, akin to Experiment 1, was implemented for all dyads.

#### Statistical Analysis

The statistical analyses followed the same procedure and used the same software as Experiment 1. One participant was excluded from the analyses due to an error rate above 20% (i.e., 319 trials). Next, 12 trials were excluded from the analyses where participants did not respond within the allotted time, and two trials were excluded from the analyses where participants responded faster than 100 ms. Finally, 947 trials were excluded from the analyses where the RTs exceeded ±2.5*SD* from the mean RT calculated per participant. The total number of excluded trials was 1280 (i.e., 3.33% of all collected trials), leaving 37104 for the statistical analysis. Of these 37104 trials, 1446 were incorrect (i.e., 3.90%).

Unlike in Experiment 1, the error rates in Experiment 2 displayed sufficient variability for statistical analysis. Therefore, the same statistical analyses described in Experiment 1 were applied to the error rates of Experiment 2. On a trial-by-trial basis, error rates are a binary outcome variable (i.e., 0 = incorrect and 1 = correct). As such, we used generalized linear mixed models (i.e., GLMM) to analyse the error rates (i.e., Binomial error distribution; Sommet & Morselli, 2017). The Wald test assessed the statistical significance of the fixed effects of the GLMM. These were the only deviations from the statistical procedure described in Experiment 1.

The full models for both ANOVA and (G)LMM analyses can be found in Supplementary file 1: Appendix F.

### Results

#### Reaction Time

**ANOVA – Neutral Participants**. A 2 × 2 repeated measures ANOVA was used to analyse median RTs of the correctly solved trials of the neutral participant with congruency (i.e., congruent and incongruent) and block type (i.e., Easy condition and Difficult condition) as independent variables. The analysis only found a significant main effect of congruency, *F*(1, 118) = 182.81, *p* < 0.001, with faster congruent trials (*M* = 492 ms) compared to incongruent trials (*M* = 527 ms), Cohen’s *d* = 0.58 (95% CI [0.49, 0.66]). No significant main effect of the block type of the manipulated participant and interaction effect were found, *p* = .165 and *p* = .908, respectively.

**ANOVA – Manipulated Participants**. The same ANOVA analysis was conducted for the manipulated participants, excluding one participant with no RTs for incongruent trials in the Easy condition. The analysis revealed a significant main effect of congruency, *F*(1, 117) = 81.14, *p* < .001, with faster congruent trials (*M* = 499 ms) compared to incongruent trials (*M* = 543 ms), Cohen’s *d* = 0.51 (95% CI [0.34, 0.67]). Moreover, there was a significant main effect of block type, *F*(1, 117) = 10.56, *p* = .002, with faster trials in the Easy condition (*M* = 512 ms) compared to the Difficult condition (*M* = 530 ms), Cohen’s *d* = 0.20 (95% CI [0.05, 0.36]). Finally, there was a significant interaction effect, *F*(1, 117) = 171.56, *p* < .001. Posthoc tests showed a congruency effect when the manipulated participants performed an Easy condition, with faster congruent trials (*M* = 456 ms) than incongruent trials (*M* = 569 ms), *t*(117) = 18.70, *p* < .001, Cohen’s *d* = 1.46 (95% CI [1.24, 1.69]). However, a reversed congruency effect was found when the manipulated participant performed a Difficult condition with slower congruent trials (*M* = 539 ms) than incongruent ones (*M* = 517 ms), *t*(117) = –2.99, *p* = .003, Cohen’s *d* = –0.25 (95% CI [–0.42, –0.08]). This observation shows that the congruency effect was reversed in the Difficult compared to the Easy condition (i.e., *Difference* = –24.2 ms and *Difference* = 113.0 ms, respectively).

**Figure 4:**
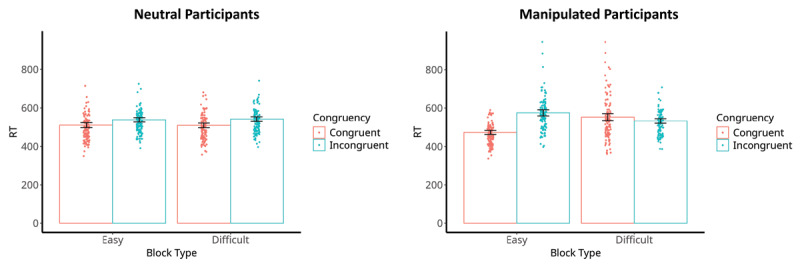
RTs on (In)Congruent Trials of the Neutral and Manipulated Participants Depending on the Difficulty of the Condition. *Note*. RT, reaction time; Easy block, block where the manipulated participant has 90% congruent trials; Difficult block, block where the manipulated participant has 10% congruent trials; left graph are the reaction times on the (in)congruent trials of the neutral participants depending on the difficulty of the condition of the manipulated participants; right graph are the reaction times on the (in)congruent trials of the manipulated participants depending on the difficulty of the condition they performed; Dots are the participants’ median RTs depending on the levels of congruency within each block type; lines on top of the bars are the 95% confidence intervals.

The LMM analyses demonstrated results similar to those of the abovementioned ANOVA analyses (see Supplementary file 1: Appendix G). Figure 4 illustrates the results of these analyses.^4^

#### Error Rates

**ANOVA – Neutral Participants**. A 2 × 2 repeated measures ANOVA was used to analyse the error rates of the neutral participant with congruency (i.e., congruent and incongruent) and block type of the manipulated participant (i.e., Easy and Difficult condition) as independent variables. The analysis showed a main effect of congruency, *F*(1, 118) = 49.95, *p* < .001, with lower error rates on congruent trials (*M* = 2.5%) compared to incongruent trials (*M* = 5.5%), Cohen’s *d* = 0.69, (95% CI [0.50, 0.87]). The main effect of block type and the interaction effect were not significant, *p* = .685 and *p* = .954, respectively.

**ANOVA – Manipulated Participants**. The same ANOVA analysis was conducted on the error rates of the manipulated participants. This analysis revealed a main effect of congruency, *F*(1, 118) = 75.53, *p* < .001, with lower error rates on congruent trials (*M* = 4.4%) compared to incongruent ones (*M* = 12.4%), Cohen’s *d* = 0.64 (95% CI [0.43, 0.85]). Moreover, the analysis revealed a significant main effect of the block type of the manipulated participant, *F*(1, 118) = 27.73, *p* < .001, with higher error rates on the Easy condition (*M* = 10.7%) compared to the Difficult condition (*M* = 6.2%), Cohen’s *d* = 0.34 (95% CI [0.16, 0.53]). Crucially, a significant interaction effect was found, *F*(1, 118) = 121.05, *p* < .001. Posthoc tests revealed a congruency effect when the manipulated participant performed the Easy condition, with lower error rates for congruent trials (*M* = 0.9%) than incongruent ones (*M* = 20.4%), *t*(118) = 11.40, *p* < .001, Cohen’s *d* = 1.41 (95% CI [1.08, 1.74]). In contrast, a reversed congruency effect was observed when the manipulated participant performed the Difficult condition, with higher error rates in the congruent (*M* = 7.9%) than in incongruent trials (*M* = 4.5%), *t*(118) = –3.51, *p* < .001, Cohen’s *d* = –0.40 (95% CI [–0.62, –0.16]). This observation shows that the congruency effect was reversed in the Difficult compared to the Easy condition (i.e., *Difference* = –3.4% and *Difference* = 19.5%, respectively).

**Figure 5:**
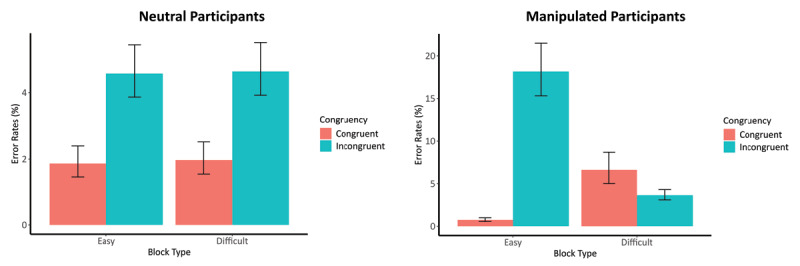
Error Rates on (In)Congruent Trials of the Neutral and Manipulated Participants Depending on the Difficulty of the Condition. *Note*. Error rates are expressed as the average percentage of error rates; Easy block, block where the manipulated participant has 90% congruent trials; Difficult block, block where the manipulated participant has 10% congruent trials; left graph are the average percentages of error rates on the (in)congruent trials of the neutral participants depending on the difficulty of the condition of the manipulated participants; right graph are the average percentages of error rates on the (in)congruent trials of the manipulated participants depending on the difficulty of the condition they performed; lines on top of the bars are the 95% confidence intervals.

The GLMM analyses demonstrated results similar to those of the abovementioned ANOVA analyses (see Supplementary file 1: Appendix G). Figure 5 illustrates the results of these analyses.^5^

## General Discussion

Across two experiments, we examined whether mental effort is contagious. To investigate this, we relied on a modified version of the joint Simon task used by Desender et al. (2016). In Experiment 1, we replicated the study of Desender et al. (2016). In Experiment 2, we adjusted the design of Desender et al. (2016) by having both participants perform a full traditional Simon task next to each other to increase the task difficulty and hence the required mental effort. In both studies, we expected manipulated participants to adapt their mental effort exertion in function of the difficulty of the blocks of trials (i.e., Easy and Difficult), and, crucially, that this mental effort adaptation would spill over to the adjacent neutral participant who performed a task that was neither Easy nor Difficult.

Both studies did not replicate the findings observed by Desender et al. (2016). Although the manipulated participants showed less pronounced differences between congruent and incongruent trials in the Difficult versus Easy blocks (i.e., PCE), their adjacent neutral participants did not adopt the same response pattern. Specifically, in both neutral blocks with an equal proportion of (in)congruent trials, the neutral participants responded faster to congruent than incongruent trials, regardless of the response pattern of the adjacent manipulated participants. In Experiment 2, we increased the mental effort required to perform the joint Simon task. Accuracy rates were considerably lower in Experiment 2 than in Experiment 1 (near ceiling, prohibiting error rate analysis), and the average Cohen’s *d* across all significant effects for the manipulated participants in Experiment 2 was larger (*d* = 0.78) than in Experiment 1 (*d* = 0.27). This suggests that the manipulated participants had to exert more mental effort in Experiment 2 than in Experiment 1. However, even with the increased mental effort required for the joint Simon task, the hypothesized contagious effect of the manipulated participant on the neutral participant was not observed. Therefore, our study provides convergent evidence from two studies that mental effort is likely not contagious.

Desender et al. (2016) originally proposed that people continuously monitor others’ behavior in everyday settings and incorporate those observations into their own cognitive control exertion. They outlined two non-mutually-exclusive mechanisms: (1) under the common-coding principle, observing subtle physical markers of effort (e.g., postural shifts) activates corresponding motor and control programs in the observer, enhancing their own effort; and (2) social-evaluative concerns may motivate observers to match others’ effort to avoid negative judgment. Our failure to replicate Desender et al.’s (2016) findings—even in Experiment 2, where the manipulated participant’s effort was clearly enhanced—sheds new light on these proposed mechanisms. It suggests that participants did not rely on social-evaluative inferences about their partner’s effort. This challenges the original claim of “mental effort contagion” and invites a reconsideration of what underlies joint task effects. Despite the absence of effort contagion, we did observe a robust congruency effect in neutral participants, even under conditions that minimize shared task representations (e.g., a separator screen, independently performed Simon task in a go/no-go fashion, and loosely aligned response keys with stimulus location; see Figure 1).

To explain this, we turn to the Theory of Event Coding (TEC; Hommel, 2019), which posits that actions and their sensory effects are integrated into multimodal event files (e.g., left press + red stimulus). Crucially, self- and other-generated actions (human or non-human) share the same event file format. For instance, Dolk et al. (2013) found that a single participant performing a joint Simon task next to a waving cat still exhibited a congruency effect, implying that the cat’s actions instantiated a competing event file that had to be distinguished from the participant’s own. In joint Simon tasks, spatial layout provides the clearest distinction—e.g., a left-hand actor versus a right-paw-waving cat. Distinguishing left- and right-hand action codes activates the corresponding motor feature codes and primes their expected sensory effects, producing interference on incongruent trials (Hommel, 1996). In our setup, observing the adjacent participant’s key presses likely instantiated such a competing event file that required spatial discrimination, producing the observed congruency effect. Exploratory evidence (see footnote 3) from Experiment 1 supports this: the neutral participant’s congruency effect was larger immediately after the manipulated participant’s trial than after their own – but only when the former was performing the Easy condition. In that Easy condition, the manipulated participant made predominantly fast congruent responses, likely making their action code especially salient in the neutral participant’s event coding (given that the neutral participant’s own task had a 50/50 congruency). This effect vanished in Experiment 2 when participants used both hands, presumably because internal representations of left- and right-hand codes preempted external influence (see footnote 4). These findings suggest a shift in how we conceptualize joint tasking. Rather than relying on shared cognitive control states or social-evaluative mechanisms, joint task effects may emerge from lower-level perceptual-motor interactions. This reframing shifts the emphasis from high-level social-cognitive mechanisms (e.g., shared goals, mental state attribution) to sensorimotor processes that operate independently of intentional coordination. In this view, joint tasking can arise even without shared goals, provided that perceptual cues are sufficiently salient to trigger event file formation.

The question remains: *why* could we not replicate the study by Desender et al. (2016)? Based on our results, we see two potential (not mutually exclusive) reasons. First, we believe a carry-over effect might have inflated or even caused the effect observed in their study. Specifically, participants who were manipulated and accustomed their mental effort in block 2 might have carried it over into block 3 when they became neutral participants. Therefore, the mental effort exerted by neutral participants in block 3 was not due to the contagious nature of the effort of the manipulated participants in block 3. However, such a carry-over effect can only be shown if the proportion of (in)congruent trials for the manipulated participants in blocks 2 and 3 dissociate (e.g., Easy versus Difficult block). This dissociation would allow observing different response patterns to (in)congruent trials in block 3 for neutral versus manipulated participants. In our exploratory analysis of Experiment 1, we observed this dissociating pattern when applying a switch transition type between blocks 2 and 3. We found, as expected, a more pronounced congruency effect in the Easy versus Difficult block for the manipulated participants in blocks 1 and 2 and 3 and 4. However, neutral participants did not show a similar effect in blocks 1 and 2 but displayed the opposite pattern in blocks 3 and 4. Specifically, they showed a more pronounced congruency effect when the manipulated participant performed the Difficult compared to the Easy block. We argue that this finding can only be explained by considering the difficulty of block 2 for the neutral participants in blocks 3 and 4. This shows that the neutral participants’ response pattern in blocks 3 and 4 is more similar to their previous response in block 2 than to the response of the adjacent manipulated participants in blocks 3 and 4. Hence, the observed pattern in blocks 3 and 4 for the neutral participants is likely driven by a carry-over effect between blocks 2 and 3. Desender et al. (2016) also analyzed blocks 1 and 2 and blocks 3 and 4 separately, observing a contagious effect in both pairs. The effect they observed in blocks 1 and 2 rule out a carry-over effect, as these blocks are free of it. However, this doesn’t exclude the possibility of a carry-over effect from block 2 to block 3, which likely inflated their findings.

Second, as we highlighted in the introduction, a false positive result is always possible (Lakens, 2022). This possibility increases when experiments observe relatively large effects with small sample sizes, which was the case for the study by Desender et al. ( \[
\eta_{p}^{2}
\] = 0.15 and *N* = 38; Lakens, 2022). Therefore, we re-analyzed their data using a more robust LMM analysis than their original ANOVA. LMM partially guards against a Type I error by accounting for random variability due to participants, block, or trial types (Barr et al., 2013). The critical takeaway is that when this variability is considered, the initial study’s effects vanish. We see this as evidence that the effect reported by Desender et al. (2016) might not have been as robust as assumed. This lack of robustness is also reflected in the relatively small effects we observed for the manipulated participants in Experiment 1 (*d* = 0.27). This means that the congruency effect was very subtle.

Third, we argue that the experimental setup of Desender et al. (2016) might not have been ideally suited for mental effort to spill over from one participant to another. To infer the mental effort of another individual, one must observe certain signs. The first set of observable signs involves physical ones (e.g., Elkins-Brown et al., 2016), mainly due to sympathetic arousal from mental effort exertion (Shenhav et al., 2017). These signs manifest themselves primarily in the face or body posture, such as increased pupil dilation (Da Silva Soares et al., 2022; van der Wel & van Steenbergen, 2018), furrowed brows, tense jaw muscles (Elkins-Brown et al., 2016; Kong et al., 2020), cardiopulmonary responses (e.g., increased respiration rate; Silva-Junior et al., 2016), and tense body postures (Friedman & Elliot, 2008). However, to pick up these physiological signs in another, one must be able to see them. In our study and Desender et al.’s (2016), participants were seated shoulder to shoulder, so they only had a clear view of each other’s hands. To observe the other participant’s face and body, they would have had to turn their heads, making it difficult to infer the other’s mental effort. We contend that mental effort might still be contagious, but this could become more evident in an experimental setup where participants can better perceive each other’s faces and bodies or even a read-out of their bodily or neural responses (cf. neural feedback). Research shows that individuals mimic others’ movements (face or body; see Cracco et al., 2018, for a meta-analysis). Therefore, if participants can pick up and mimic the physiological signs of mental effort, it is reasonable to assume they might also infer this effort and experience similar levels of mental effort. One approach is to place the participants on opposite sides of the table facing each other, letting them perform the task on a tablet with antiglare so they cannot see each other’s screens. A second set of observable signs relates to observing the task performance of the adjacent participants (Dolk et al., 2013). However, as already shown in Experiment 1 of Desender et al. (2016, cf. footnote 1), observing each other’s task performance allows the neutral participant to predict whether the upcoming response is congruent or incongruent. Hence, the separator screen was implemented in Experiment 2 of Desender et al. (2016) and our Experiments 1 and 2.

In conclusion, our two studies found no evidence that mental effort is contagious between participants, failing to replicate Desender et al. (2016). While manipulated participants showed expected effort differences based on task difficulty, neutral participants did not mirror these patterns. The neutral participants did exhibit a congruency effect even under conditions that minimized shared task representations. We therefore suggest that joint tasking can arise without shared goals or cognitive states, provided that perceptual-motor cues are sufficiently salient to trigger event file formation. Moreover, we also argued that the discrepancy in results with Desender et al.’s original study suggests that a carry-over effect or other methodological issues may have influenced the original study’s effect. Our re-analysis using a robust LMM approach indicated that Desender et al.’s (2016) findings might not be as robust as initially conceived. The experimental setup, which limited participants’ visibility of each other’s physiological signs and performance, likely also hindered their ability to infer each other’s mental effort and mimic behaviour. Future studies should improve the visibility of participants’ faces and bodies to test this effect more accurately. Our findings underscore the need for robust experimental design and analysis in psychological research. While our studies do not support mental effort contagion, they offer insights for refining future research approaches on this topic.

## Data Accessibility Statement

The preregistrations of the hypotheses, sample size, and statistical analysis, along with the data, code, and Supplementary file 1 appendices, are available on the Open Science Framework via this link: https://osf.io/486vn/.

## Footnotes

Desender et al. (2016) conducted two experiments. The first experiment was like a traditional Simon task but performed by two participants without using a separator screen. The authors detected a potential response bias for the neutral participant with this specific setup. If the manipulated participant performed a Difficult condition, most of their target stimuli would appear on the side of the screen of the neutral participant (i.e., 90% incongruent trials). This also means that the target stimuli of the neutral participant would have a higher likelihood of appearing on the manipulated participants’ side of the screen (50% incongruent trials) than those of the manipulated participant (i.e., 10% congruent trials). As such, the neutral participant could have possibly anticipated their incongruent target stimuli. So, instead of the neutral participant being influenced by the mental effort exertion of the manipulated participant in the Difficult condition, they might just have adopted a response strategy to cope with the task (see Desender et al., 2016, for a detailed discussion). This pattern is the same for the Easy condition, but then reversed. Therefore, we solely focused on study two of Desender et al. (2016).

Due to a technical issue with the online recruitment platform, the age of 58 participants were not registered.

We also conducted a sequential, trial-by-trial adaptation analysis to examine whether mental effort spills over from one participant to the adjacent participant based on who the actor was on the previous trial (see Yamaguchi et al., 2018). An LMM on RTs with congruency (congruent and incongruent), previous actor (same and different), block type of the manipulated participant (Easy and Difficult condition), and current actor (neutral and manipulated) as independent variables revealed a significant four-way interaction, χ^2^(1) = 9.87, *p* = .002 (see Supplementary file 1: Appendix E, for a detailed description of the results). Follow-up tests showed that both the neutral and manipulated participants only responded in line with their respective Simon task context, as indicated by a congruency effect for the neutral participant and a PCE for the manipulated participant. For the neutral participant, the congruency effect, in the Easy condition only, was more pronounced after the manipulated participant’s performance (Δ 19 ms, *p* < .001, Cohen’s *d = 0.28*) than their own (Δ 11 ms, *p* < .001, Cohen’s *d* = 0.15), likely reflecting simple motor priming from their partner’s movement dynamics. For the manipulated participant, the PCE was more pronounced following their own performance (Difficult condition: Δ -4 ms, *p* = .146, Cohen’s *d* = 0.05) than that of the neutral participant (Difficult condition: Δ 9 ms, *p* < .001, Cohen’s *d* = 0.13). This finding is likely due to the brief interruption in one’s own responding while the other participant responded, partially resetting the cognitive control adaptation. These findings reinforce our general conclusion in the main analyses that adjacent partners do not co-represent each other’s mental effort exertion, while demonstrating that simple motor priming and inter-trial interruptions (trials where the other participant is the actor) likely influence sequential modulations (See also General Discussion).

We also conducted a sequential, trial-by-trial adaptation analysis on RTs in Experiment 2 (see Yamaguchi et al., 2018). An LMM with congruency (congruent and incongruent), previous actor (same and different), block type of the manipulated participant (Easy and Difficult condition), and current actor (neutral and manipulated) as the independent variables showed a significant four-way interaction, χ²(1) = 7.47, *p* = .006 (see Supplementary file 1: Appendix H, for a detailed description of the results). Follow-up tests showed that neutral participants’ congruency effects were equally large regardless of block type and who acted previously (Easy condition: same Δ 29 ms and different Δ 28 ms; Difficult condition: same Δ 32 ms and different Δ 35 ms)— unlike in Experiment 1, where they dissociated in the Easy condition. Manipulated participants again exhibited a stronger PCE when following their own performance (Difficult condition: Δ –29 ms, *p* < .001, Cohen’s *d* = –0.29) than that of the neutral participant (Difficult condition: Δ –10 ms, *p* = .088, Cohen’s *d* = –0.09). In line with Experiment 1, we attribute this pattern of results to brief interruptions in one’s own responding when the adjacent partner acted, partially resetting participants’ cognitive control adaptation. As in Experiment 1, these results reinforce our main conclusion that adjacent partners do not co-represent each other’s mental effort.

We also conducted a sequential, trial-by-trial adaptation analysis on the error rates in Experiment 2 (see Yamaguchi et al., 2018). A GLMM with congruency (congruent and incongruent), previous actor (same and different), block type of the manipulated participant (Easy and Difficult condition), and current actor (neutral and manipulated) as the independent variables showed a significant four-way interaction, χ²(1) = 5.01, *p* = .025 (see Supplementary file 1: Appendix H, for a detailed description of the results). Follow-up tests mirrored the pattern for RTs of Experiment 2: neutral participants’ error rate congruency effects were equally large regardless of previous actor identity and block type (Easy condition: same Δ 2.2% and different Δ 2.5%; Difficult condition: same Δ 2.5% and different Δ 2.9%), whereas manipulated participants exhibited a stronger PCE when following their own performance (Difficult condition: Δ –2.7%, *p* = .006, Cohen’s *d* = –0.84) than that of the neutral participant (Difficult condition: Δ 1.7%, *p* = .152, Cohen’s *d* = –0.32).
